# Prevalence and Correlates of Video and Internet Gaming Addiction among Hong Kong Adolescents: A Pilot Study

**DOI:** 10.1155/2014/874648

**Published:** 2014-06-16

**Authors:** Chong-Wen Wang, Cecilia L. W. Chan, Kwok-Kei Mak, Sai-Yin Ho, Paul W. C. Wong, Rainbow T. H. Ho

**Affiliations:** ^1^Centre on Behavioral Health, The University of Hong Kong, Sassoon Road, Pokfulam, Hong Kong; ^2^Department of Social Work & Social Administration, The University of Hong Kong, Pokfulam, Hong Kong; ^3^School of Public Health, The University of Hong Kong, Sassoon Road, Pokfulam, Hong Kong

## Abstract

This pilot study investigated the patterns of video and internet gaming habits and the prevalence and correlates of gaming addiction in Hong Kong adolescents. A total of 503 students were recruited from two secondary schools. Addictive behaviors of video and internet gaming were assessed using the Game Addiction Scale. Risk factors for gaming addiction were examined using logistical regression. An overwhelming majority of the subjects (94%) reported using video or internet games, with one in six (15.6%) identified as having a gaming addiction. The risk for gaming addiction was significantly higher among boys, those with poor academic performance, and those who preferred multiplayer online games. Gaming addiction was significantly associated with the average time spent gaming per week, frequency of spending money on gaming, period of spending money on gaming, perceived family disharmony, and having more close friends. These results suggest that effective educational and preventative programs or strategies are needed.

## 1. Introduction

With the popularity of high-tech devices (computer, tablet, and smartphone) and internet use in recent years, playing online or offline games has become a popular activity, especially among young people. People usually play video games for entertainment, excitement, challenge seeking, emotional coping, and escaping from reality to virtuality to fulfill their unsatisfied needs or motivations [1]. Although some studies have demonstrated beneficial effects of playing video games on psychological and physical health [2, 3], most research on video games has focused on the negative effects on gamers. It has been suggested that excessive video gaming is associated with reduced sleep time, limited leisure activities, insomnia [4], attention problems, poor academic performance [5], anxiety, depressive symptoms, deterioration of interpersonal relationships, family conflicts, youth violence or crimes [1], lower self-esteem, and lower satisfaction with daily life [6]. Video game addiction may lead to particularly serious health problems in adolescents as they are experiencing significant physical and psychosocial changes and lack self-regulation [7–9]. Worldwide, addiction to video games is becoming a serious concern among youth players [10].

During the past decade, a number of studies have examined internet addiction in general among adolescents with various diagnostic criteria and the results were inconsistent [7, 9, 11–13]. However, the term “internet addiction” is not endorsed in the recently published fifth edition of the* Diagnostic and Statistical Manual of Mental Disorders (DSM-5)*. Instead, the term “internet gaming disorder” has been included in the third section of the* DSM-5* as a condition warranting more clinical research and experience before it might be considered a formal disorder [14]. Thus, scientific studies on internet or video gaming addiction are indeed needed.

The number of studies in this field is limited and different terms, such as problematic computer game use [15], problematic video game use [16–18], video game dependency [4], and pathological video game use [5, 19, 20], have been used. Agreement is lacking on the criteria of “problematic use,” which is a general term and usually milder than “pathological use.” However, the term “pathological” is not endorsed in the* DSM-5* to avoid reinforcing the social stigma of being a problem user [21]. In the present study, we use the term “gaming addiction” as a synonym of “pathological use” to refer to a condition whereby functional impairment in daily life is caused by excessive video or internet gaming.

A few studies have examined the prevalence of video gaming addiction [4, 5, 15–17, 19, 20, 22, 23], but the reported data varied greatly across countries. Choo et al. reported a prevalence of 8.7% among Singaporean youth [20]. Salguero and Morán found a similar rate of 9.9% among Spanish adolescents [18]. A study using a national sample of American youth aged 8 to 18 years indicated a prevalence of 8.0% [5]. Rehbein and colleagues [4] found among German adolescents a prevalence of 1.7%, but another study [15] reported a lower prevalence of 0.2%. Haagsma et al. reported a prevalence of 3.3% among adolescents and young adults in the Netherlands [16]. The inconsistent findings may be explained by several factors, especially sociocultural and criteria differences [24]. To our knowledge, no studies in this field have been conducted in Hong Kong.

Given the vulnerability of adolescents who are facing developmental challenges, a good understanding of video gaming activities among this group would be helpful for tailoring effective education or prevention programs to promote their health. Thus, the first objective of this study was to examine the profiles of video gaming behaviors in adolescents. The second objective was to estimate the prevalence of gaming addiction and examine its correlates in a sample of Hong Kong adolescents.

## 2. Methods

### 2.1. Subjects and Procedure

This cross-sectional study was conducted in October 2013. Subjects were recruited mainly from two secondary schools in different districts (Central District and Kowloon East) of Hong Kong. The schools were randomly selected and all students in the selected classes of different grades, namely, grades 8 to 11, were invited to participate in the survey. Students in the first year of secondary school (grade 7) were not contacted due to recent transition in their school life. Students in the last year of high school (grade 12) were also not contacted due to their study load. Each subject completed an anonymous questionnaire. The purposes of the study were fully introduced, and school, parental, and student consents were obtained before data collection. Of 520 students invited, 503 (96%) returned a valid questionnaire. Ethical approval was obtained from the Human Research Ethics Committee for Non-Clinical Faculties of the University of Hong Kong.

### 2.2. Measurements

#### 2.2.1. Social and Demographic Information

Demographic and personal information obtained included age, sex, grade, number of close friends, and self-rated levels of stress and loneliness. Family-related factors included parental education level and marital status, family economic status, perceived family harmony, and ownership of devices including computer, tablet, and smartphone. School-related factors included self-rated academic performance, relationship with classmates, and relationship with teachers. Family harmony was assessed by a revised version of the Family Harmony Index [25], which included five items to assess the quality of relationships with or between family members (3 items), students' obedience to their parents (1 item), and perceived care from their parents (1 item) on a 5-point Likert scale ranging from 0 (never) to 4 (very often). The scale score is the sum of all items. Cronbach's alpha for the scale was 0.83 in the present study. Each student indicated the number of friends they could talk to about private matters and the number of friends they could call on for help. The number of close friends was the average of these two numbers. Other factors were measured using single items.

#### 2.2.2. Game Playing

First, respondents were asked to report how many days they usually played games each week during the past 6 months and how many hours they played on an average weekday and on weekend days. Total playing time per week was estimated by multiplying the hours played on a typical weekday by the number of weekdays that the respondent reported playing plus the total playing time on weekend days. Second, respondents were asked about the type of game (e.g., multiplayer online games, online single player games, and offline casual games) they most frequently played. Third, they were asked to report the frequency of spending money on playing games per month during the past year using three items to assess how often they bought DVD games, played commercial games, and spent money to play online games on a 5-point Likert scale ranging from 0 (never) to 4 (very often). They were also asked to report the total amount of money they had spent on playing games in an average month (one item). Finally, they were asked to indicate how long they had been playing internet games and how long they had been spending money on playing games.

#### 2.2.3. Problematic Behaviors

Problematic behaviors of video and internet gaming were assessed by a translated Chinese version of the short form of the Game Addiction Scale (GAS) [26], which was developed to assess the extent of gaming-related problems among adolescents. A systematic review of psychometric assessment tools for pathological video gaming suggests that the GAS provides the most clinically relevant information and has demonstrated strong convergent validity [27]. The scale includes seven items to assess seven core components of video gaming addiction (preoccupation/salience, withdrawal symptoms, tolerance, problems, conflict, loss of interest, and mood modification), which are consistent with the symptom criteria for internet addiction as identified in a previous study [24]. The symptom criteria have been used as the key* DSM-5* criteria for internet gaming disorder [14]. Subjects were asked to indicate the frequency with which they had experienced each of the described situations over the past 6 months on a five-point Likert scale ranging from 1 (never) to 5 (very often). These items were derived from the original 21-item scale, based on the highest factor loading. Although the full version may be more reliable and nuanced, the short form of the GAS seems to be psychometrically sound and useful for large-scale surveys [15]. The internal consistency of the short form of the GAS was good with Cronbach's alpha of 0.93 and 0.94 for the two schools in the present study. A factor analysis of all seven items in the GAS provided support for a single-factor model.

According to Lemmens et al. [26], ratings of 3 (sometimes) or above in GAS items denoted game addiction symptoms. A monothetic approach wherein respondents have to endorse all seven items to identify gaming addiction and a polythetic approach wherein respondents endorse at least four of the items to categorize problematic gamers were suggested in the original study [26]. In accordance with the key potential criteria (i.e., two core symptoms (preoccupation and withdrawal symptoms) plus at least one of the five other symptoms) for internet gaming disorder [14, 24], we used a modified polythetic approach to screen for gaming addiction, requiring that at least 3 GAS items had a rating of 4 (often) or above. Compared to the monothetic approach recommended by Lemmens et al. [26], the modified approach demonstrated the same sensitivity (81.0%) and an improved specificity (84.6% versus 87.3%) in the present study.

Problematic behaviors of internet use in general were assessed by the internet addiction test (IAT) [28]. The IAT is a widely used scale in the field and has been validated in the traditional Chinese version [29]. It includes 20 items derived from the* DSM-IV-R* diagnostic criteria for pathological gambling and rated on a Likert scale from 1 (rarely) to 5 (very often). The maximum score for the scale is 100 and a score of 70 or above indicates internet addiction [28]. In the present study, the IAT was used to validate the GAS, since playing online games was a powerful predictor of internet addiction [13]. Subjective awareness of gaming-related problems was also assessed by two items asking students if their time management has been affected by playing games and if their study or homework has been affected by playing games on a five-point Likert scale ranging from 0 (never) to 4 (very often).

## 3. Statistical Analysis

Descriptive analyses were used to describe the students' social and demographic information, gaming behaviors, and the prevalence of probable gaming addiction. Student's *t*-test and chi-square test were used to examine sex difference for continuous variables and categorical variables, respectively. Hierarchical logistic regression analyses were performed to examine the predictive power of different variables for gaming addiction, with demographics and family- and school-related variables put into the regression model in the first step, the types of games in the second step, and gaming behavior related variables in the third step. In order to reduce the number of potential covariates with low explanatory power, bivariate correlation analyses were performed before regression analyses and only statistically significant variables were selected. All statistical analyses were conducted using the IBM SPSS Statistics (20.0) software. A *P* value of less than 0.05 was considered statistically significant.

## 4. Results

### 4.1. Social and Demographic Characteristics

The demographic characteristics and family- and school-related variables are displayed in Table 1. There was no significant difference between boys and girls except for the number of close friends (*P* < 0.001) and ownership of computer (*P* < 0.01).

### 4.2. Gaming Behaviors

#### 4.2.1. Playing Frequency and Playing Time

Of all subjects, 46% played games almost every day and 47.2% played some days a week. On weekdays, 22.9% played more than 3 hours and 31.2% played more than 1 hour each day, while on weekend days 36.6% played more than 3 hours and 32% played more than 1 hour a day. Around 21% of respondents downloaded games, 6% bought DVD games, 7% played commercial games on casino machines, and 11% spent money to play online games often or very often. In total, around 40% reported having spent money on gaming, of which 3.6% spent more than HK$500 (US$65) and 9.9% spent HK$200–500 (US$25–64) each month. Around 29% had been spending money to play games for more than one year.

#### 4.2.2. Differences in Playing Frequency and Playing Time According to Demographics

More boys (54%) than girls (38%) reported playing games almost every day. Only 4% of boys and 9.5% of girls did not play games regularly (*P* = 0.001). During weekdays, 27.3% of boys played more than 3 hours and 35.1% played 1 to 3 hours per day, compared with only 18.5% and 27.3% in girls, respectively (*P* = 0.001). During weekend days, more boys (44.3%) than girls (29.2%) played games more than 3 hours per day (*P* < 0.001). In boys, 59% had been spending money on gaming and 5.6% had spent more than HK$500 (US$65) each month, which were more common than that of 21.7% and 1.6%, respectively, in girls (*P* < 0.001). Generally, more boys than girls had been spending money on buying DVD games (*P* < 0.001), playing commercial games (*P* < 0.001), and playing online games (*P* < 0.001). More boys than girls had been spending money to play games for more than one year (*P* < 0.001). No significant differences in gaming behaviors by grade were observed except that lower grade students spent more time playing games during weekdays (*P* = 0.005) and more frequently spent money on DVD games (*P* = 0.042).

#### 4.2.3. Types of Games Played

In total, 46.7% of the subjects preferred playing online multiplayer games, 16.9% preferred playing online single player games, and only 10% preferred playing offline casual games. More boys (69.1%) than girls (24.8%) preferred playing online multiplayer games, whereas more girls (24%) than boys (9.6%) preferred playing online single player games (both *P* < 0.001). Although nonsignificant (*P* = 0.059), fewer grade 11 students played games than lower grades.

### 4.3. Problematic Gaming Behavior

#### 4.3.1. Prevalence of Probable Gaming Addiction

The percentages of respondents who have endorsed the criteria often and very often for each item of the GAS are presented in Table 2. Based on the modified criteria for the GAS, 15.7% of the students met the criteria for probable gaming addiction. The rate was significantly higher in boys (22.7%) than girls (8.7%) (*P* < 0.001).

Given that the Chinese version of the GAS has not been validated before, we have also examined validity of the GAS. Results based on Pearson correlation analyses indicated that the composite score of the GAS correlated strongly with the subjects' awareness of the problem for time management (*r* = 0.814, *P* < 0.001) and academic performance (*r* = 0.817, *P* < 0.001) affected by playing games. The composite score of the GAS was also significantly correlated with the total score of the IAT (*r* = 0.619, *P* < 0.001).

#### 4.3.2. Correlates of Gaming Addiction with Social and Demographic Variables

Bivariate correlation analysis showed that gaming addiction was related to sex, academic performance, perceived family harmony, classmate relations, and the number of close friends but not related to age, grade, number of family members/siblings, parental marital status, parental education, family economic status, and ownership of devices (computer, tablet, and smartphone). The results of multivariate logistic regression analysis (Table 3) indicated that gaming addiction was significantly more likely in boys (OR = 2.49, 95% CI: 1.41–4.40), those with poor academic performance (OR = 2.80, 95% CI: 1.13–6.92), those with perceived family disharmony (OR = 3.36, 95% CI: 1.53–7.41), and those who reported more friends than others (OR = 3.08, 95% CI: 1.63–5.82).

#### 4.3.3. Correlates of Gaming Addiction with Gaming Habits

As shown in Table 3, the risk for gaming addiction was significantly higher among those who preferred multiplayer online games (OR = 2.50; 95% CI: 1.29–4.58) than those who preferred other games. Bivariate correlation analysis indicated that gaming addiction was correlated with the average time of gaming per week, frequency of spending money on gaming, amount of money spent on gaming, period of playing internet games, and period of spending money on gaming. After putting these variables into the regression model and adjusting for other variables, gaming addiction was significantly associated with the longer average time of gaming per week (OR = 1.32, 95% CI: 1.16–1.49), higher frequency of spending money on gaming (OR = 1.18, 95% CI: 1.02–1.36), and longer period of spending on gaming (OR = 1.35, 95% CI: 1.01–1.81), but not with the amount of money spent on gaming and period of playing internet games.

## 5. Discussion

The present study is probably the first to examine the pattern of video or internet gaming habits in particular among Hong Kong adolescents. The results showed that playing video games was a widespread activity among Hong Kong adolescents. Few adolescents (7%) did not play games regularly. Our results also showed that around 40% of Hong Kong adolescents had been spending money on gaming regularly, although the amount of money was limited, and that boys spent more time and more money on gaming than girls. For the large majority of adolescents, playing games appeared to be a harmless leisure activity. Previous studies have suggested that moderate use of the internet and computer games is associated with a more positive academic orientation than nonuse or high levels of use [23]. However, a small proportion of gamers with overuse of video and internet games might have shown problematic gaming behaviors.

Currently, there is still a lack of culturally sensitive instrument to screen problematic gaming behaviors in Chinese society. In this study we used the GAS, which was developed in the Netherlands, to estimate the prevalence of gaming addiction. Psychometric properties of the scale have been examined in this study and our results have shown good reliability and validity for the Chinese version of the GAS. In the present study, we have used a revised scoring approach for GAS. Our results suggest that the revised scoring approach seems better than the original approach. Lemmens et al. [26] suggest that the criteria for each item must be fulfilled at least occasionally. This definition should be discussed critically and used cautiously. According to the* DSM-5* criteria, symptoms for internet gaming disorder should last at least 3 months [14, 24]. Thus, the chosen cut-off threshold is relatively low and gaming addiction would be overestimated [15]. Thus, it may be more reliable to use the revised approach proposed in the present study to identify probable gaming addiction.

We found that 15.6% of the respondents could be considered as probably addicted gamers. This prevalence appears to be higher than that reported in other regions [4, 5, 16, 18, 20] but comparable to the reported rates of internet addiction in general in Hong Kong as reported in previous studies [7, 12, 13, 30]. An earlier study reported that roughly 20% of adolescents aged 11–18 years could be classified as internet addicts [13]. Another study suggested that 17.2% of students were addicted to the internet [30]. In a recent study, Shek and Yu reported that 26.7% of early adolescents met the defined criteria for internet addiction [7]. Hong Kong is a densely populated city where the space for outdoor physical activities is very limited and a sedentary lifestyle dominates. Playing video or internet games may be a major form of recreation for a number of adolescents. Thus, a high rate of gaming addiction may be understandable. The observed rate of affected adolescents points out a need for effective education and prevention programs or strategies in Hong Kong to avoid negative effects of video gaming on adolescents.

In our study, a significant difference in the prevalence of gaming addiction between boys and girls has been observed, which is consistent with the results of recent studies on gaming addiction [4–6, 16, 20] but inconsistent with the results of previous studies on internet addiction in general [7, 9]. The disparity may be attributable to the different online activities of males and females. Usually, boys prefer to play video and internet games, while girls prefer to use the internet for social media [31]. A meaningful finding in this study may be that perceived family disharmony is significantly associated with gaming addiction in adolescents. Generally, family plays a very important role in the psychosocial development and well-being of children, especially in Chinese societies. Previous studies have suggested that high parent-adolescent conflict [32] or conflictive family relations [9] predict internet addiction in adolescents. Our results further indicate that playing video games may be a major online activity for those adolescents, since playing games may help them to forget about or “escape” unpleasant things, reduce tension, and improve mood [33]. The results of previous studies may highlight the importance of family intervention for internet addiction in adolescents, and the results of the present study may help to improve the efficiency of family intervention. On the other hand, it is also likely that addicted behaviors in adolescents may lead to family disharmony. Further longitudinal studies are needed to address the causal relationship between family harmony and video or internet gaming addiction. Consistent with previous reports [9], we did not find an association between gaming addiction and parents' education level.

Our results showed that those students who reported having more close friends were more likely to demonstrate gaming addiction than those who reported fewer friends. This finding is consistent with the results of a study on problematic internet use in Chinese students [9]. The result may be ascribed to peer effect, since adolescents who are addicted to gaming tend to interplay with more friends on the internet. Previous studies have indicated that peer relationships have a strong positive effect on substance use in adolescents [34]. To date, studies on the effects of peer influence on gaming behaviors are still limited. Further research is needed to explore the pattern of interplay with peers on the internet among adolescents. With regard to school-related factors, our study indicated that students with poor school performance had a higher risk for gaming addiction. This is consistent with previous findings [4, 5, 20]. Contrary to previous reports on internet addiction in general [9, 35, 36], we did not find an association between gaming addiction and stress level or poor classmate relationships, possibly due to the small sample size. Further large-scale studies may generate meaningful findings in this aspect.

In line with earlier findings [4, 16, 37, 38], our results indicated that those who played multiplayer online games played for more hours per week and had higher risk for gaming addiction, possibly due to increased enjoyment and interplay with other players, which might result in prolonged gaming. We also noted that the time spent on gaming was positively correlated with gaming addiction. This is also consistent with the results of previous studies [16, 19]. Therefore, restricting adolescents' time for gaming may be an effective measure to prevent gaming addiction. A possible unique and interesting finding of this study is that the risk for gaming addiction is significantly associated with the period and frequency of spending money on gaming, but not with the amount of money spent on gaming. To our knowledge, few studies have addressed this issue before. Unlike online gambling among adults, adolescents usually spend money on gaming for fun rather than for profit. However, persistent spending of money on gaming may be an important predictor of gaming addiction. Our findings may have implications for intervention.

Our results should be interpreted in light of several limitations. First, the focus of the present study was on video and internet gaming in general rather than internet gaming in particular. To date, studies on internet gaming are still limited. A focus on video gaming in general makes our results comparable to the reports of previous studies. Second, the present study was not based on structured psychiatric interview and diagnostic criteria for internet gaming disorder [39], but this did not undermine the reliability and implications of our findings. We aimed to screen adolescents at high risk. An attention to the at-risk group is always important for prevention of clinical problems. Third, the cross-sectional research design of the present study may not allow causal conclusions between video gaming addiction and relevant factors. Moreover, a modest sample size in this pilot study may make robust estimates difficult, but this does not diminish the statistical significance of our results. However, it should be cautious to generalize the reported rates in our study due to the pilot study design. Further large-scale studies may be warranted. Finally, only the short form of the GAS was used and validated in this study. There may be a concern about whether some items from the GAS can reliably discriminate problematic behavior from healthy and enthusiastic behaviors in the Chinese cultural context. Further validation of the Chinese version of the 21-item GAS is needed. Despite these limitations, the current study is among the first to examine video gaming habits among adolescents in a modern Chinese society and provides a useful addition to the literature related to addictive behaviors.

In conclusion, playing video and internet games is a widespread activity among Hong Kong adolescents, and a substantial proportion of adolescents may exhibit addictive behaviors with regard to video and internet games. Special attention should be paid to those students who are particularly vulnerable to video and internet gaming addiction. Given that adolescence is a time in which people experience significant biological, psychological, and social changes, effective education and intervention programs may be needed to help adolescents and youths to successfully navigate the developmental challenges. The correlated factors identified in the present study may highlight the importance of family-targeted and school-based education or prevention programs in this aspect. Further research is needed to understand the underlying mechanisms of video and internet gaming addiction and to explore effective preventative or interventional strategies.

## Figures and Tables

**Table 1:** Comparison of sociodemographic and family- and school-related variables between boys and girls.

	Girls		Boys		*r* or *x* ^2^ values	*P* values
	M	SD	M	SD		

Age	14.54	1.42	14.62	1.35	0.598	0.550
	N	%	N	%		
Subjects	254	50.5	249	49.5		
Grade					1.29	0.732
Grade 8	71	28.7	60	25.1		
Grade 9	81	30.0	81	33.9		
Grade 10	47	19.0	43	18.0		
Grade 11	55	22.3	55	23.0		
Academic performance					2.163	0.339
Good or very good	51	20.2	43	17.3		
Fair	162	64.3	155	62.5		
Poor or very poor	39	15.5	50	20.2		
Marital status of parents					1.214	0.545
In marriage	203	80.9	193	78.5		
Separated or divorced	42	16.7	43	17.5		
One parent passed away	6	2.4	10	4.1		
Father′s education					0.88	0.644
Primary school level	35	14.6	37	15.6		
Secondary school level	186	77.8	177	74.7		
College level or above	18	7.5	23	9.7		
Mother′s education					0.077	0.962
Primary school level	42	17.2	40	16.9		
Secondary school level	187	76.6	181	76.4		
College level or above	15	6.1	16	6.8		
Family′s economic status					7.52	0.111
Rather wealthy	2	0.8	3	1.2		
Medium wealthy	28	11.4	30	12.2		
Medium	170	69.1	143	58.4		
Medium low	36	14.6	57	23.3		
Rather poor	10	4.1	12	4.9		
Perceived family harmony					0.216	0.897
Very good or good	111	43.9	104	42.3		
General	114	45.1	112	45.5		
Bad or very bad	28	11.1	30	12.2		
Teacher relations					2.147	0.342
Very good or good	157	62.5	164	66.9		
General	83	33.1	67	27.3		
Bad or very bad	11	4.4	14	5.7		
Classmate relations					3.614	0.164
Very good or good	194	77.3	171	70.1		
General	51	20.3	63	25.8		
Bad or very bad	6	2.4	10	4.1		
Number of close friends					16.563	<0.001
2 or fewer	79	31.5	71	28.7		
3 to 6	152	60.6	125	50.6		
7 or more	20	8	51	20.6		
Stress level					2.199	0.333
Much	54	21.3	57	23.1		
A little	147	58.1	128	51.8		
None	52	20.6	62	25.1		
Loneliness level					1.663	0.435
Much	35	13.8	43	17.4		
A little	121	47.6	119	48.2		
None	98	38.6	85	34.4		
Ownership of computer					9.489	0.009
Personal use	95	37.8	99	40.2		
Shared with others	154	61.4	134	54.5		
None	2	0.8	13	5.3		
Ownership of tablet					0.059	0.971
Personal use	38	15.6	37	14.9		
Shared with others	68	27.9	69	27.7		
None	138	56.6	143	57.4		
Use of mobile phone					7.337	0.062
Smartphone	238	93.7	220	88.4		
Ordinary mobile phone	9	3.5	12	4.8		
None	3	1.2	13	5.2		

**Table 2:** Percentages of responses (often and very often) for each item of the Game Addiction Scale.

	Often		Very often		Total	
	*n *	%	*n *	%	*n *	%
(i) Did you think about playing a game all day long?	58	11.5	38	7.6	96	19.1
(ii) Did you spend increasing amounts of time on games?	52	10.3	36	7.2	88	17.5
(iii) Did you play games to forget about real life?	34	6.8	34	6.8	68	13.5
(iv) Have others unsuccessfully tried to reduce your game use?	37	7.4	40	8.0	77	15.3
(v) Have you felt bad when you were unable to play?	32	6.4	24	4.8	56	11.1
(vi) Did you have fights with others (e.g., family, friends) over your time spent on games?	30	6.0	32	6.4	62	12.3
(vii) Have you neglected other important activities (e.g., school, work, sports) to play games?	34	6.8	32	6.4	66	13.1

**Table 3:** Logistic regression analysis of the risk for gaming addiction (total *R* ^2^ = 0.39).

		OR	95% CI	*P* values	*R* ^ 2^ change
Step 1	Sex				0.16
	Girls	1.00			
	Boys	2.49	1.41–4.40	0.002	
	Academic performance				
	Good or very good	1.00			
	Fair	1.55	0.70–3.45	0.458	
	Poor or very poor	2.80	1.13–6.92	0.026	
	Family harmony				
	Good or very good	1.00			
	General	1.15	0.62–2.12	0.654	
	Bad or very bad	3.36	1.53–7.41	0.003	
	Classmate relations				
	Good or very good	1.00			
	General	1.04	0.55–1.96	0.897	
	Bad or very bad	1.37	0.40–4.70	0.620	
	Number of close friends				
	6 or less	1.00			
	7 or more	3.08	1.63–5.82	0.001	
Step 2	Types of games				0.02
	Other types of video games	1.00			
	Multiplayer online games	2.50	1.29–4.85	0.007	
Step 3	Average time of gaming per week	1.32	1.16–1.49	<0.001	0.21
	Frequency of spending money on gaming	1.18	1.02–1.36	0.025	
	Amount of money spent on gaming per month	0.92	0.62–1.37	0.696	
	Period of spending money on gaming	1.35	1.01–1.81	0.042	
	Period of playing internet games	1.03	0.75–1.41	0.873	
